# A Case of Uterine Hydatids

**Published:** 1858-12

**Authors:** James B. McCaw


*A Case of Uterine Hydatids.* Reported by James B. McCaw, M.D.

The following sketch of a case of cystic degeneration of the chorion occurred in my practice some years ago, and will be found not without interest to the reader:

Mrs. F. had been married about fifteen years, and had given birth to eight healthy and well formed children. She had always carried her children with great ease, and the periods of pregnancy and of parturition had been entirely natural.

In the year 1851 she again conceived. The menstrual secretion disappeared, and for three months there were no unusual symptoms, save probably more morning sickness and rather more irritability of bladder than was common in the previous conceptions.

About the close of the third month, this lady was suddenly seized with a large hemorrhage, as she supposed, and her physician was hastily called in. I found her, on my arrival, in bed, and her clothes drenched with a fluid which was like currant juice and water in color; but the amount of discharge was so great as to satisfy me that the larger portion of it must be water, although there certainly had been considerable loss of blood. Her pulse was fluttering, and there were some symptoms of swooning. The discharge, however, had ceased; and although no hydatid membranes were discernible, I concluded that the discharge of fluid must be attributable to this cause.

In about three weeks after this occurrence the patient had another attack of like character with the one just mentioned. The discharge was more serous, not so large, and there was less constitutional disturbance. The abdominal walls were unnaturally distended prior to both of these attacks, and she evidently diminished greatly in size after they passed off. I could trace no membranous appearances on this occasion, and was rather disposed to conclude that this was a case of dropsy of the membranes and not of hydatid formations.

In the sixth month of pregnancy I was sent for in haste to visit this lady, and found her positively in labor, although she was not disposed to believe it, because she had never felt her child. The os tincæ was dilating, and there was a good deal of blood passing. She was very large, and the uterine th[illegible]s were quick and active.

As she was losing more blood than was safe, I tamponed the vagina with a sponge, and waited the progress of events. In about an hour the sponge was expelled, and the breach of a withered, half-grown fœtus presented itself at the vulva, and was easily expelled. The next instant, and with a great flow of bloody water, a mass of serous bags which were commingled with a growth evidently of a placental character, and wrapped up in the uterine membranes, was driven out with force, and a good deal of hemorrhage. The uterus contracted well, however, and the loss of blood was not very great. The degenerated mass of placenta, hydatids and membranes filled a wash-basin nearly.

An examination showed that a partial placenta had been formed, which I suppose was sufficient to nourish up to a certain point the halt-developed fœtus; but the larger portion of this mass consisted of hydatid cysts seeming to be growing out of the chorion at the point where the imperfect placenta was attached, and indeed intimately connected with it.

This case satisfied me that hydatid growths in the uterus were to be regarded as a morbid development of the chorion, which, instead of resulting in the formation of a natural placenta, degenerated into a conglomeration of serous cysts, hanging together like grapes, and filled with watery fluid. It also would lead to the conclusion that such morbid growths were always the result of conception, and could never, as some authors think, occur in the virgin womb.

It is difficult to say whether we should believe in the theory laid down by most of the pathological anatomists, that these hydatids were independent organisms of low vitality, parasites of the body, and not morbid growths of the animal structure. The microscopists contend that an inspection of these membranous bags exhibit the peculiar conformation of the *acephalocyst*, and hence they regard them as of parasitic origin. I am rather disposed to go with Ashwell, Meigs, and the practical obstetricians, who consider them as degenerated conditions of the uterine and placental membranes, springing up during the progress of pregnancy, and interfering with the proper development of the fœtus.—*Virginia Medical Journal.*

